# The impact of the COVID-19 pandemic on sleep disorders among Nursing professionals^1^

**DOI:** 10.1590/1518-8345.6043.3795

**Published:** 2023-03-06

**Authors:** Carla Renata Silva Andrechuk, Juliano de Souza Caliari, Mariana Alvina dos Santos, Flávia Helena Pereira, Henrique Ceretta Oliveira, Maria Filomena Ceolim

**Affiliations:** 1 Universidade Estadual de Campinas, Faculdade de Enfermagem, Campinas, SP, Brazil.; 2 Instituto de Educação, Ciência e Tecnologia do Sul de Minas Gerais, Passos, MG, Brazil.; 3 Universidade Federal de Mato Grosso do Sul, Três Lagoas, MS, Brazil.

**Keywords:** Nurse Practitioners, Nursing, Sleep, COVID-19, Pandemics, Health Surveys, Profissionais de Enfermagem, Enfermagem, Sono, COVID-19, Pandemias, Inquéritos Epidemiológicos, Enfermeras Practicantes, Enfermería, Sueño, COVID-19, Pandemias, Encuestas Epidemiológicas

## Abstract

**Objective:**

to analyze the factors related to sleep disorders reported by Nursing professionals during the COVID-19 pandemic.

**Method:**

this is a cross-sectional and analytical study conducted with Nursing professionals from all Brazilian regions. Sociodemographic data, working conditions and questions about sleep disorders were collected. The Poisson regression model with repeated measures was used to estimate the Relative Risk.

**Results:**

572 answers were analyzed, which revealed that non-ideal sleep duration, poor sleep quality and dreams about the work environment were predominant during the pandemic, with 75.2%, 67.1% and 66.8% respectively; as well as complaints of difficulty sleeping, daytime sleepiness and non-restorative sleep during the pandemic were reported by 523 (91.4%), 440 (76.9%) and 419 (73.2%) of the Nursing professionals, respectively. The relative risk of having such sleep disorders during the pandemic was significant for all variables and categories studied.

**Conclusion:**

non-ideal sleep duration, poor sleep quality, dreams about the work environment, complaints regarding difficulty sleeping, daytime sleepiness and non-restorative sleep were the predominant sleep disorders among Nursing professionals during the pandemic. Such findings point to possible consequences on health, as well as on the quality of the work performed.

## Resumo

**Objetivo:**

analisar os fatores relacionados às alterações no sono relatadas pelos profissionais de enfermagem durante a pandemia de COVID-19.

**Método:**

trata-se de um estudo transversal e analítico, realizado com profissionais de enfermagem de todas as regiões do Brasil. Foram coletados dados de caracterização sociodemográfica, condições de trabalho e questões sobre alterações de sono. Para estimar o Risco Relativo foi utilizado o modelo de regressão de Poisson com medidas repetidas.

**Resultados:**

foram analisadas 572 respostas, as quais revelaram que a duração não ideal do sono, a má qualidade do sono e os sonhos com o ambiente de trabalho foram predominantes durante a pandemia, com 75,2%, 67,1% e 66,8% respectivamente, assim como as queixas de dificuldade ao dormir, sonolência diurna e sono não restaurador durante a pandemia foram relatadas por 523 (91,4%), 440 (76,9%) e 419 (73,2%) dos profissionais de enfermagem, respectivamente. O risco relativo de apresentar tais alterações de sono, durante a pandemia foi significativo para todas as variáveis e as categorias estudadas.

**Conclusão:**

duração não ideal do sono, má qualidade do sono, sonhos com o ambiente de trabalho, queixas de dificuldade ao dormir, sonolência diurna e sono não restaurador foram as alterações do sono predominantes entre os profissionais de enfermagem durante a pandemia. Estes achados apontam para possíveis consequências na saúde, bem como na qualidade do trabalho realizado.

## Resumen

**Objetivo:**

analizar los factores relacionados con los trastornos del sueño que informaron los profesionales de enfermería durante la pandemia de COVID-19.

**Método:**

se trata de un estudio transversal y analítico realizado con profesionales de enfermería de todas las regiones de Brasil. Se recolectaron datos sobre caracterización sociodemográfica, condiciones de trabajo y preguntas sobre trastornos del sueño. Para estimar el Riesgo Relativo se utilizó el modelo de regresión de Poisson con medidas repetidas.

**Resultados:**

se analizaron 572 respuestas, que revelaron que durante la pandemia predominaron la duración del sueño no ideal, la mala calidad del sueño y los sueños sobre el ambiente laboral, con 75,2%, 67,1% y 66,8% respectivamente, además 523 (91,4%), 440 (76,9%) y 419 (73,2%) profesionales de enfermería manifestaron quejas de dificultad para conciliar el sueño, somnolencia diurna y sueño no reparador durante la pandemia, respectivamente. El riesgo relativo de padecer trastornos del sueño durante la pandemia fue significativo para todas las variables y categorías estudiadas.

**Conclusión:**

la duración del sueño no ideal, la mala calidad del sueño, los sueños sobre el ambiente laboral, las quejas de dificultad para conciliar el sueño, la somnolencia diurna y el sueño no reparador fueron los trastornos del sueño predominantes en los profesionales de enfermería durante la pandemia. Estos hallazgos indican posibles consecuencias para la salud, así como para la calidad del trabajo realizado.

**Highlights**

(1) The results pointed to a high risk of sleep disorders.

(2) Sleep disorders can compromise health and quality of care.

(3) There is a need to implement measures to improve well-being in the workplace.

(4) There is a need for measures to reduce stress among Nursing professionals.

(5) There is an urgent need for interventions that improve the Nursing professionals’ sleep.

## Introduction

Coping with COVID-19, a disease caused by the *Severe Acute Respiratory Syndrome Coronavirus 2* (SARS-CoV-2), can lead to the emergence of physical and emotional problems such as anxiety, fear, depressive symptoms and changes in the circadian cycle, with disturbances in the sleep pattern^1^. 

Sleep quality is intrinsically related to neurobehavioral functions and to balance of the immune system, being considered one of the mechanisms to reduce contamination by opportunistic diseases^2^ ^-^ ^3^. 

In health professionals, the impacts of the pandemic are enhanced by physical exhaustion, high workload, lack of personal protective equipment, inadequate working conditions, need for ethically complex decisions on care rationing, emotional tensions and severity of the cases^4^. Such occupational demands are frequent causes of stress and insufficient sleep and can contribute to changes in the work process, increasing the risk of accidents^5^.

High stress levels and poor sleep quality are among the effects observed in Nursing professionals^5^. In a study conducted with 2,372 nurses, it was observed that 69.3% presented habitual sleep deficits and that the lower the mindfulness level and the worse the work/family conflict, the worse the sleep quality^6^. Thus, the improvement in sleep satisfaction, duration and efficiency can promote benefits for well-being and for the quality of Nursing care^7^.

The recommendations for adequate sleep-related quality and duration are important tools for the public health context. Due to the importance of sleep in several health and well-being aspects, the National Sleep Foundation (2020)^8^ published the importance of sleep and guidelines for sleeping well during the COVID-19 pandemic.

A number of experts warn that, during the pandemic, people started to have more dreams and remember them more easily, in addition to reporting that they often involved images and unpleasant situations. More disturbed sleep can increase the probability of worrisome dreams, in turn causing stress and anxiety, which impair sleep quality^8^. Given this context, this research was guided by the hypothesis that the COVID-19 pandemic may have negatively affected the sleep of Nursing professionals working in health care.

Considering the importance of sleep throughout the life and work dynamics, this study aims at analyzing the factors related to sleep disorders reported by Nursing professionals during the COVID-19 pandemic.

## Method

### Type of study

This is a cross-sectional and analytical study, based on Strengthening the Reporting of Observational studies in Epidemiology (STROBE)^9^.

### Locus

It was conducted in all Brazilian regions.

### Period

June and July 2020.

### Population

Nursing professionals who worked in health care.

### Selection criteria

The inclusion criteria adopted were the following: age greater than or equal to 18 years old and whether the Nursing professional was working in health care during the COVID-19 pandemic. Workers exclusively working in the night shift were excluded.

### Definition of the sample

Sample calculation was performed in order to estimate the proportion of participants with sleep disorders. For this purpose, the reference was the population of Nursing professionals in the country: 2,304,509^10^, with 5% sampling error and 99% confidence level. A proportion of 0.5 was considered, whose value represents the maximum variability of the binomial distribution, which generated an estimate with the largest possible sample size^11^. For this study, the estimated sample size was 664 participants. 

### Study variables

The variables of interest in the study were as follows: identification of the Nursing professional, gender (men, women), professional training (nursing assistant, nursing technician, nurse), number of work contracts (one, two or more), weekly hour load (20-30 hours, 31-40 hours, 41-50 hours, more than 50 hours) and health performance levels (primary and secondary, tertiary, pre-hospital). Also in relation to sleep, some variables were built by asking the following questions before the pandemic: “How many hours of sleep did you use to sleep per night”, “Which difficulties did you experience when sleeping?”, “Did you feel sleepy during the day?”, “Did you use to have dreams about the work environment?”, “Did you wake up willing after sleep?”, “How did you classify your sleep?” and the below questions during the pandemic: “How many hours of sleep do you sleep *per* night?”, “Which difficulties do you experience when sleeping?”, “Do you feel sleepy during the day?”, “Do you usually have dreams about the work environment?”, “Do you wake up willing after sleep?”, “How do you classify your sleep?” and “Have you started using sleep medications?”. According to the answers, the following categories were considered for analysis: ideal sleep duration (7-8 hours) and non-ideal sleep duration (less than or equal to 6 hours or greater than or equal to 09 hours)^12^; poor sleep quality (poor and very poor answers) and good sleep quality (very good and good answers); there were two possible answers for all the other variables: Yes or No. 

### Instruments used for data collection

An online questionnaire was used for sociodemographic characterization, working conditions and sleep disorders, built and submitted to content validation by four judges, with recognized knowledge in the areas of sleep and validation of measuring instruments.

### Data collection

The study was conducted through an online questionnaire, self-completed through a computer or mobile phone with Internet access. The questionnaire was prepared using the Google Forms app. The “snowball” chain sampling procedure adapted to virtual social networks was used^13^. At the beginning of the study, invitations were sent with the link to access the questionnaire through the Facebook and Instagram virtual social networks, WhatsApp or e-mail to an initial group of people who are part of the target population (called “seeds”), which indicate peers from the same population group and so on.

### Data treatment and analysis

The data were exported from Google Forms directly to a Microsoft Office Excel^®^ spreadsheet, double-checked, with removal of incomplete questionnaires and then transferred to the Statistical Analysis System software (version 9.4, Stata Corp LLC, College Station, United States). Data treatment was performed using descriptive statistics (frequency, percentage, central tendency and dispersion measures) and the modified Poisson regression model was used to estimate the Relative Risk, with robust variance, for repeated^14^ and simple measures, as well as for those with an interaction effect, with presentation of their respective 95% confidence intervals and p-values with the significance level set at 5% (p<0.05). 

### Ethical aspects

The study was submitted and approved by the Research Ethics Committee, under Certificate of Presentation of Ethical Appraisal (*Certificado de Apresentação de Apreciação* Ética, CAAE) No. 31681020.9.0000.8158, issued on May 29^th^, 2020. Its development complied with the ethical precepts set forth in Resolution No. 466/2012 of the National Health Council.

## Results

A total of 577 answers to the survey were received; however, 572 Nursing professionals who met the inclusion criteria were considered to comprise the sample. Description of the sample evidenced a majority of women (88.8%) and a mean age of 36.4 years old (SD=8.8). In relation to the work variables, most of them were nurses (71.5%), reporting only one employment contract (69.2%), working in tertiary care (68.9%) and having an hour load of 31-40 hours a week (45.8%). The characteristics of the sample profile are presented in Table 1.

**Table 1:** Sociodemographic and work characterization of the Nursing professionals (n=572). Brazil, 2020

Variables	n	%
Gender		
Female	508	88.8
Male	64	11.2
Professional training		
Nursing Assistant	14	2.5
Nursing Technician	149	26.0
Nurse	409	71.5
Number of work contracts		
One	396	69.2
Two or more	176	30.8
Weekly hour load (hours)		
20 - 30	69	12.1
31 - 40	262	45.8
41 - 50	115	20.1
50+	126	22.0
Health performance levels		
Primary and secondary	123	21.5
Tertiary	394	68.9
Pre-hospital	55	9.6

The study revealed that, among the Nursing professionals, 71.8% reported good sleep quality before COVID-19; during the pandemic and social distancing period, this percentage dropped to only 32.9%. There was a reduction in the mean sleep duration in the pandemic (5h 46 min, SD=1.30) when compared to the period before the pandemic (7h 07 min, SD=1.36) (data not shown in the tables).

It was verified that non-ideal sleep duration, its poor quality and dreams about the work environment were predominant in the pandemic for all the variables investigated and in all categories (Table 2).

**Table 2:** Description of the sociodemographic and work-related variables, and changes reported during the COVID-19 pandemic as a function of sleep duration, sleep quality and dreams about the work environment (n=572). Brazil, 2020

Variables	Non-ideal sleep duration		Poor sleep quality		Dreams about the work environment	
	Before the pandemic n(%)	During the pandemic n(%)	Before the pandemic n(%)	During the pandemic n(%)	Before the pandemic n(%)	During the pandemic n(%)
Total number of participants	247 (43.2)	430 (75.2)	161 (28.2)	384 (67.1)	231 (40.4)	382 (66.8)
Gender						
Female	215 (42.2)	375 (73.8)	139 (27.4)	345 (67.9)	203 (40.0)	350 (68.9)
Male	32 (50.0)	55 (85.9)	22 (34.4)	39 (60.9)	28 (43.8)	32 (50.0)
Professional training						
Nursing Assistant	8 (57.4)	11 (78.8)	6 (42.9)	12 (85.7)	5 (35.7)	11 (78.6)
Nursing Technician	63 (42.3)	118 (79.2)	40 (26.9)	103 (69.1)	53 (35.6)	103 (69.1)
Nurse	176 (43.0)	301 (73.6)	115 (28.1)	269 (65.8)	173 (42.3)	268 (65.5)
Number of work contracts						
One	157 (39.6)	280 (70.7)	105 (26.5)	253 (63.9)	151 (38.1)	272 (68.7)
Two or more	90 (51.1)	150 (85.2)	56 (31.8)	131 (74.4)	80 (45.5)	110 (62.5)
Weekly hour load (hours)						
20 - 30	27 (39.1)	51 (73.9)	19 (27.5)	40 (58.0)	30 (43.5)	40 (58.0)
31 - 40	100 (38.2)	186 (71.0)	69 (26.3)	158 (60.3)	98 (37.4)	176 (67.2)
41 - 50	55 (47.8)	84 (73.0)	36 (31.3)	83 (72.2)	46 (40.0)	84 (73.0)
50+	65 (51.6)	109 (86.5)	37 (29.4)	103 (81.7)	57 (45.2)	82 (65.1)
Started using sleep medications						
No	183 (43.2)	310 (73.1)	103 (24.3)	257 (60.6)	173 (40.8)	263 (62.0)
Yes	64 (43.2)	120 (81.1)	58 (39.2)	127 (85.8)	58 (39.2)	119 (80.4)

Complaints regarding difficulty sleeping, daytime sleepiness and non-restorative sleep were more expressive during the pandemic, being reported by 523 (91.4%), 440 (76.9%) and 419 (73.2%) of the Nursing professionals, respectively. Table 3 shows the sociodemographic and work-related variables, as well as the changes reported during the COVID-19 pandemic, as a function of the sleep complaints. 

**Table 3:** Description of the sociodemographic and work-related variables, as well as changes reported during the COVID-19 pandemic as a function of the sleep complaints (n=572). Brazil, 2020

Variables	Difficulty sleeping		Daytime sleepiness		Non-restorative sleep	
	Before the pandemic n(%)	During the pandemic n(%)	Before the pandemic n(%)	During the pandemic n(%)	Before the pandemic n(%)	During the pandemic n(%)
Total number of participants	423 (74.0)	523 (91.4)	282 (49.3)	440 (76.9)	267 (46.7)	419 (73.2)
Gender						
Women	373 (73.4)	464 (91.3)	245 (48.2)	390 (76.8)	231 (45.5)	373 (73.4)
Men	50 (78.1)	59 (92.2)	37 (57.8)	50 (78.1)	36 (56.2)	46 (71.9)
Professional training						
Nursing Assistant	12 (85.7)	14 (100%)	4 (28.6)	7 (50.0)	7 (50.0)	10 (71.4)
Nursing Technician	119 (79.9)	141 (94.6)	61 (40.9)	121 (81.2)	71 (47.6)	105 (29.5)
Nurse	292 (71.4)	368 (90.0)	217 (53.1)	312 (76.3)	189 (46.2)	304 (74.3)
Number of work contracts						
One	294 (74.2)	368 (92.9)	191 (48.2)	294 (74.2)	175 (44.2)	276 (69.7)
Two or more	129 (73.3)	155 (88.1)	91 (51.7)	146 (83.0)	92 (52.3)	143 (81.2)
Weekly hour load (hours)						
20 - 30	52 (75.4)	66 (95.7)	31 (44.9)	48 (69.6)	34 (49.3)	49 (71.0)
31 - 40	195 (74.4)	235 (89.7)	124 (47.3)	197 (75.2)	124 (47.3)	179 (68.3)
41 - 50	86 (74.8)	107 (93.0)	55 (47.8)	87 (75.7)	51 (44.3)	86 (74.8)
50+	90 (71.4)	115 (91.3)	72 (57.1)	108 (85.7)	58 (46.0)	105 (83.3)
Started using sleep medications						
No	300 (70.8)	376 (88.7)	216 (50.9)	316 (74.5)	194 (45.7)	294 (69.3)
Yes	123 (83.1)	147 (99.3)	66 (44.6)	124 (83.8)	73 (49.3)	125 (84.5)

The relative risk of reporting non-ideal sleep duration, poor quality sleep and dreams about the work environment during the pandemic, when compared to the period before it, was significantly higher for all participants, as well as for almost all the categories of sociodemographic and work-related variables analyzed (Table 4).

**Table 4:** Poisson regression for the risk of non-ideal sleep duration, poor sleep quality and dreams about the work environment (n=572). Brazil, 2020

Variables	Non-ideal sleep duration			Poor sleep quality			Dreams about the work environment		
	RR^*^	95% CI	p-value^‡^	RR^*^	95% CI	p-value^‡^	RR^*^	95% CI	p-value^‡^
**Total number of participants (after *vs*. before)**	1.74	1.58 - 1.91	<0.01	2.39	2.11 - 2.69	<0.01	1.65	1.47 - 1.86	<0.01
Gender									
Men (after *vs*. before)	1.72	1.37 - 2.15	<0.01	1.77	1.30 - 2.41	<0.01	1.14	0.77 - 1.69	0.51
Women (after *vs*. before)	1.74	1.57 - 1.93	<0.01	2.48	2.18 - 2.83	<0.01	1.72	1.52 - 1.95	<0.01
Professional training									
Assistant (after *vs*. before)	1.38	0.86 - 2.19	0.18	2.00	1.14 - 3.52	0.02	1.90	1.04 - 4.65	0.04
Technician (after *vs*. before)	1.71	1.53 - 1.91	<0.01	2.34	2.03 - 2.70	<0.01	1.55	1.35 - 1.78	<0.01
Nurse (after *vs*. before)	1.87	1.53 - 2.29	<0.01	2.58	2.02 - 3.28	<0.01	1.94	1.51 - 2.50	<0.01
Number of work contracts									
One (after *vs*. before)	1.67	1.44 - 1.93	<0.01	2.34	1.91 - 2.87	<0.01	1.38	1.12 - 1.69	<0.01
Two or more (after *vs*. before)	1.78	1.58 - 2.01	<0.01	2.41	2.07 - 2.80	<0.01	1.80	1.56 - 2.08	<0.01
Weekly hour load (hours)									
20 - 30 (after *vs*. before)	1.89	1.44 - 2.47	<0.01	2.11	1.50 - 2.96	<0.01	1.33	0.95 - 1.87	0.10
31 - 40 (after *vs*. before)	1.86	1.59 - 2.17	<0.01	2.29	1.91 - 2.75	<0.01	1.80	1.49 - 2.17	<0.01
31 - 50 (after *vs*. before)	1.53	1.25 - 1.86	<0.01	2.31	1.78 - 2.98	<0.01	1.83	1.41 - 2.36	<0.01
50+ (after *vs*. before)	1.68	1.41 - 2.00	<0.01	2.78	2.15 - 3.60	<0.01	1.44	1.15 - 1.81	<0.01
Started using sleep medications									
Yes (after *vs*. before)	1.88	1.55 - 2.27	<0.01	2.19	1.79 - 2.68	<0.01	2.05	1.65 - 2.54	<0.01
No (after *vs.* before)	1.69	1.52 - 1.89	<0.01	2.50	2.14 - 2.90	<0.01	1.52	1.32 - 1.75	<0.01

^*^RR = Relative Risk estimated through the Poisson regression model with repeated and simple measures, as well as those with interaction effect; ^†^CI = Confidence Interval; ^‡^p-value = Significance level

When analyzing the sleep complaints (difficulty sleeping, daytime sleepiness and non-restorative sleep), there was a significantly higher risk for them to be reported during the pandemic, when compared to the period before it for all participants, as well as for almost all the different categories of sociodemographic and work-related variables analyzed (Table 5).

**Table 5:** Poisson regression for the risk of difficulty sleeping, daytime sleepiness, and non-restorative sleep (n=572). Brazil, 2020

Variables	Difficulty sleeping			Daytime sleepiness			Non-restorative sleep		
	RR^*^	95% CI	p-value^‡^	RR^*^	95% CI	p-value^‡^	RR^*^	95% CI	p-value^‡^
**Total number of participants (after *vs*. before)**	1.24	1.18-1.29	<0.01	1.56	1.42-1.71	<0.01	1.57	1.44-1.71	<0.01
Gender									
Men (after *vs*. before)	1.18	1.06-1.31	<0.01	1.35	1.06-1.73	0.02	1.28	1.03-1.58	0.03
Women (after *vs*. before)	1.24	1.19-1.30	<0.01	1.59	1.44-1.76	<0.01	1.61	1.47-1.77	<0.01
Professional training									
Assistant (after *vs*. before)	1.17	0.94-1.44	0.16	1.75	0.92-3.32	0.09	1.43	0.77-2.66	0.26
Technician (after *vs*. before)	1.26	1.19-1.33	<0.01	1.44	1.29-1.60	<0.01	1.61	1.46-1.77	<0.01
Nurse (after *vs*. before)	1.18	1.10-1.28	<0.01	1.98	1.61-2.44	<0.01	1.48	1.25-1.75	<0.01
Number of work contracts									
One (after *vs*. before)	1.20	1.12-1.29	<0.01	1.60	1.38-1.86	<0.01	1.55	1.37-1.77	<0.01
Two or more (after *vs*. before)	1.25	1.19-1.32	<0.01	1.54	1.37-1.73	<0.01	1.58	1.41-1.76	<0.01
Weekly hour load (hours)									
20 - 30 (after *vs*. before)	1.27	1.11-1.45	<0.01	1.55	1.15-2.09	<0.01	1.44	1.17-1.78	<0.01
31 - 40 (after *vs*. before)	1.21	1.13-1.28	<0.01	1.59	1.37-1.85	<0.01	1.44	1.28-1.63	<0.01
31 - 50 (after *vs*. before)	1.24	1.13-1.37	<0.01	1.58	1.27-1.96	<0.01	1.69	1.38-2.07	<0.01
50+ (after *vs*. before)	1.28	1.16-1.41	<0.01	1.50	1.29-1.74	<0.01	1.81	1.51-2.17	<0.01
Started using sleep medications									
Yes (after *vs*. before)	1.20	1.11-1.29	<0.01	1.88	1.55-2.28	<0.01	1.71	1.46-2.01	<0.01
No (after *vs*. before)	1.25	1.19-1.32	<0.01	1.46	1.32-1.63	<0.01	1.52	1.37-1.67	<0.01

^*^RR = Relative Risk estimated through the Poisson regression model with repeated and simple measures, as well as those with interaction effect; ^†^CI = Confidence Interval; ^‡^p-value = Significance level

## Discussion

Working in health care during the COVID-19 pandemic caused several physical and psychological disorders in many professionals, especially nursing assistants, nursing technicians and nurses. 

The results described confirm the impact of the pandemic on sleep disorders and corroborate other studies that point to variables such as gender^15^ ^-^ ^16^, professional training^4^, number of work contracts, weekly hour load^1^ ^,^ ^16^ ^-^ ^17^ and having started using sleep medications^4^, related to sleep disorders.

Harmful outcomes in the sleep quality of the Nursing professionals^4^ who work directly with people affected by COVID-19 have been reported, which can be related to increased stress in the work environments and to the impact on the habitual sleep patterns^5^ ^,^ ^17^. 

The risk for sleep disorders during the pandemic was significant for both genders in all variables, except in relation to the dreams about the work environment, where only the women remained statistically significant. In the relationship between the gender variable and sleep disorders, being a woman has always been associated with greater chances of having sleep disorders when compared to men, which corroborates a national study carried out with 45,160 individuals aged 18 years old and over, in which 51.1% of the participants were women and presented an increase or incidence of sleep problems during the pandemic, when compared to the men (37.7%) with the same problems^16^. 

Being a woman increased the chance for deterioration in terms of sleep problems during the pandemic. They tended to present worse health conditions and higher work overload due to double or triple workdays, which can be more incident during this period, especially in relation to household chores^16^ ^,^ ^18^.

In an Italian study conducted with Nursing professionals who treated COVID-19 patients, it was identified that being a woman was associated with higher anxiety and sleep deprivation levels and that gender was a predictive factor for increased anxiety and poor sleep quality^19^.

During the pandemic, nursing technicians and nurses presented a significant risk of developing all the sleep disorders investigated in this study, while for the professional assistants, only the risk of having poor sleep quality and dreams about the work environment was significant. The significant increase in the prevalence of sleep disorders among Nursing professionals can be related to the increased stress they faced during the treatment of COVID-19 patients^20^.

In a cross-sectional study conducted with 1,257 health professionals in 34 hospitals from China, the Nursing professionals reported more severe degrees of all the measurements of depression, anxiety, insomnia and distress symptoms than other health professionals, pointing out that the professionals working on the front line were at a high risk of developing changes in their health, requiring immediate care^18^.

In relation to the number of work contracts, although the association was statistically significant for the “one” and “two or more” categories, it was noticed that having “two or more contracts” was related to a higher chance of presenting sleep disorders, which can be explained by the increased workload and the fear of infecting people at their homes. 

During the COVID-19 pandemic, in addition to working long hours with a high risk of infection, Nursing professionals sometimes also faced situations of lack of medical supplies and personnel, due to illness in their colleagues, which contributes to anxiety and stress^6^, thus affecting sleep quality.

In a study carried out in Turkey, 81.4% of the nurses reported that their co-workers were infected by COVID-19 and 92.6% stated they were afraid of infecting people in their homes and such results are linked to increased stress scores and related to poor sleep patterns^5^. Likewise, despite being statistically significant in all categories, the relationship with the weekly hour load corroborates that the predominance of 50 hours or more, with a higher chance of presenting sleep disorders, points to the impact of work activities in the sleep profile, which can be linked to increased stress, suggesting that sleep problems are common in health professionals who experience high stress levels at work^5^.

In a study carried out by the Oswaldo Cruz Foundation in partnership with the State University of Campinas and the Federal University of Minas Gerais, it was identified that, in the general population, the high volume of activities in the domestic routine resulting from the pandemic increased sleep disorders by two times. Such being the case, the higher number of work activities might increase the time required to perform all the daily tasks, depriving people of sleep time or impacting on stress and anxiety, thus contributing to worsen sleep quality^1^ ^,^ ^16^.

With the pandemic and the increase in work overload, tension in the work environment increased and the hours of sleep decreased. In a national study conducted with the general population, the results showed that 67% of the people had a change in their sleep routine, with some individuals sleeping more hours a day and others less^21^. In another study conducted in the Philippines with clinical nurses, it was found that low scores on the pandemic fatigue scale were related to better sleep quality and to greater job satisfaction. It is essential that institutional measures are in place to address this issue in the professionals and to promote their health and overall well-being^22^.

With the increase in the users’ demand for health services, in addition to the distancing of contaminated professionals, of those without due physical conditions and of an age not compatible with the recommendations of health bodies to act in the fight against COVID-19, an increase in hours and care demands was triggered, favoring stress of the work environment as a reason for the active professionals to sleep less and worse^17^, which may have been intensified with the increase in the number of work contracts.

Another important result of this study was verified for the “started using sleep medications” variable, noting that in both categories, “yes” and “no”, there was a significant risk for presenting all of the sleep disorders under study during the pandemic, although those who reported having started using such medications were at a greater risk of reporting such changes. A number of studies show that the emergence of the new coronavirus can trigger many psychosocial problems, including sleep disorders, which may justify the use of sleep medications during the pandemic^1^ ^,^ ^23^. 

It is known that sleep disorders can be the result of malfunctions in various regulatory mechanisms. Insomnia, for example, is a multidimensional condition that reflects the changes in physical, mental and emotional aspects, representing a difficulty initiating, maintaining and consolidating sleep or deterioration in the overall sleep quality, generating or aggravating the physical and mental harms^24^. In addition, low quality in the sleep pattern might also contribute to accidents and risky behaviors, causing failures in the care provided, as well as harms to the patient and the institution^25^. Thus, the real meaning of these effects of impaired sleep on the lives of the affected professionals must be taken into account, in addition to the associations evidenced in the current research, such as the feelings awakened in a person who used to enjoy satisfactory sleep and started to have no more.

The cross-sectional design can be pointed out as a limitation of this study, since no causal association can be made as in randomized controlled clinical studies, in addition to the sample size reaching 86.2%. The second is representativeness of the population, as there was a need for access and familiarity with Internet use to answer the questionnaires. The third is the fact that the participants may have answered the questions during or after a stressful workday, which may have influenced the result, interfering with proper judgment of the changes that occurred during the pandemic. The fourth is in the variables investigated, with the possibility of other factors such as mental health disorders, use of licit and illicit drugs and other types of medication that act on the Central Nervous System, among others, that can negatively affect sleep quality and that were not investigated in this study. Finally, the use of an instrument exclusively elaborated for the study since, despite being extremely useful and valuable, the instruments validated for use in Brazil did not cover all the aspects that were sought to be evaluated. 

This study has the following strengths: the fact that it covered all regions of the country, with participants who worked on the front line of the fight against COVID-19, with high levels of occupational stress, fear and uncertainties. Assessing sleep among Nursing professionals in this setting, given that it was at the beginning of the pandemic, signaled changes in various aspects related to sleep, being extremely relevant to assess the impact on their health.

## Conclusion

The results clearly point out that, during the pandemic, sleep disorders were predominant (non-ideal sleep duration, poor sleep quality, dreams about the work environment, complaints regarding difficulty sleeping, daytime sleepiness and non-restorative sleep). There was an increase in the prevalence of such changes for all the variables investigated and in almost all categories. The relative risk for presenting such changes during the pandemic was also verified for all the variables under study. 

The data showed the urgent need for interventions that can minimize the risks caused by COVID-19, especially in the sleep of the professionals who work on the front line in the fight against this disease, which is a concern to maintain the quality of the Nursing care offered.
